# Soft Tissue Cephalometric Analysis of the Lips in Orthodontic Diagnosis and Treatment Planning: A Narrative Review

**DOI:** 10.7759/cureus.113704

**Published:** 2026-07-31

**Authors:** Pooja Kapoor, Harpreet Singh

**Affiliations:** 1 Department of Orthodontics and Dentofacial Orthopaedics, Government Dental College and Hospital, Patiala, IND; 2 Department of Conservative Dentistry and Endodontics, Desh Bhagat Dental College and Hospital, Mandi Gobindgarh, IND

**Keywords:** cephalometry, facial aesthetics, lip posture, orthodontics, soft tissue analysis

## Abstract

Facial aesthetics and orthodontic treatment planning depend not only on hard tissue management but also on the careful handling of surrounding soft tissues. Hence, meticulous evaluation of the lip position, posture, strain, and prominence is of paramount significance. Various soft tissue cephalometric analyses have been described in the literature, and their optimal and selective application can help achieve improved treatment outcomes, particularly in the management of complex orthodontic cases. This narrative review summarizes commonly used soft tissue cephalometric analyses of the lips with special emphasis on their clinical significance, advantages, and limitations.

## Introduction and background

Facial aesthetics (FA) is one of the primary reasons patients seek orthodontic treatment [1]. Successful orthodontic treatment requires a comprehensive approach that integrates both hard and soft tissue considerations. Accurate diagnosis and optimal treatment planning necessitate a meticulous assessment of skeletal, dental, and soft tissue analyses (STA). Of the various soft tissue components, the lips are critical determinants of facial attractiveness, smile aesthetics, and profile balance. Historically, cephalometric analyses (CA) have focused predominantly on hard tissue relationships and skeletal landmarks [2]. However, it is now recognized that skeletal correction alone does not always translate into optimal FA. The presence of lip protrusion, retrusion, incompetence, or strain despite an ideal skeletal relationship may compromise treatment outcomes. Lip position is not only influenced by the underlying skeletal framework but also by ethnicity, age, gender, muscular tonicity, and dental inclination [3,4].

This underscores the importance of integrating optimal soft tissue cephalometric analyses (STCA) with conventional hard tissue analyses (HTA) to achieve balanced and harmonious orthodontic outcomes. Over the decades, several STCA have been introduced [5-7]. These analyses utilize specific anatomical landmarks to assess lip position relative to the nose, chin, and overall facial profile. Advances in digital orthodontics have further enhanced their precision and clinical applicability [8,9]. This narrative review aims to provide an overview of the various STCA used for lip profile assessment and to emphasize their role in contemporary orthodontic diagnosis and treatment planning (ODTP).

Literature search strategy

A narrative review of the literature was conducted using the electronic databases PubMed, Scopus, and ScienceDirect. Relevant publications published in English between 1950 and 2025 were identified using combinations of the following keywords: soft tissue cephalometric analysis, lip posture, lip prominence, orthodontic soft tissue analysis, facial aesthetics, cephalometric lip analysis, and orthodontic profile assessment. Additional relevant articles were identified through the reference lists of selected publications where appropriate. Original research articles, review papers, and relevant textbooks focusing on soft tissue evaluation in orthodontics were included to provide a comprehensive overview of the evolution, applications, and clinical significance of STCA.

Lip anatomy

The lips, anatomically termed the labium superius oris (upper lip) and labium inferius oris (lower lip), form the anterior boundary of the oral cavity and are essential components of FA and oral function. Each lip consists of three distinct regions: the external cutaneous surface, the vermilion border, and the internal mucosal lining. Although aesthetic evaluations commonly focus on the vermilion, the anatomical extent of the lips is much broader. The upper lip extends from the base of the nose and the nasolabial folds to the vermilion border, whereas the lower lip spans from one oral commissure to the other and continues inferiorly to the labiomental crease. The upper and lower lips meet laterally at the oral commissures, which serve as important attachment sites for several muscles responsible for lip movement and facial expression.

The vermilion represents a specialized transition zone between the external skin and the oral mucosa. Histologically, it is composed of a thin, non-keratinized stratified squamous epithelium that is only three to five cell layers thick, making it considerably thinner than the surrounding facial skin, which typically contains approximately 16 layers. Unlike the cutaneous portion of the lips, the vermilion is devoid of hair follicles, sweat glands, sebaceous glands, and salivary glands. Its characteristic coloration varies from pinkish-red to brown depending on an individual's ethnicity and skin pigmentation. The distinctive reddish appearance results from the minimal presence of melanocytes combined with an abundant superficial vascular network, allowing underlying blood vessels to be readily visible, particularly in individuals with lighter skin tones.

The boundary separating the dry vermilion from the moist oral mucosa is known as the red line. Beyond this junction, the wet labial mucosa contains numerous minor salivary glands within the submucosal tissue, and the surface markings characteristic of the external lip disappear. This transition reflects the change from the external environment to the internal lining of the oral cavity [10-14].

Several anatomical landmarks of the lips contribute significantly to facial attractiveness. Among these, the philtrum is a key determinant of upper lip aesthetics, with ideal dimensions reported to be approximately 10-11 mm in width and 12-15 mm in length. As part of the normal aging process, the philtrum progressively elongates while the upper vermilion becomes thinner. Consequently, philtral length may increase from the youthful range of 12-15 mm to approximately 18-20 mm, contributing to the characteristic age-related changes observed in the perioral region. Another important aesthetic landmark is Cupid's bow, the central portion of the upper mucocutaneous junction located at the base of the philtral columns. Although defects in Cupid's bow may be concealed by facial hair in males, loss or flattening of this landmark in females often results in a less defined and aesthetically flattened upper lip [15].

Beyond their cosmetic significance, the lips perform several indispensable physiological functions. They facilitate mastication by maintaining food within the oral cavity and creating an effective seal that prevents the escape of liquids during eating and drinking. In infants, the lips are crucial for breastfeeding, as they form a tight seal around the nipple to generate the negative pressure required for effective suckling.

Lip length and incisor coverage

The mean upper lip length typically ranges from 18 to 22 mm [16]. Differences in upper lip length have been reported between males and females, with males typically exhibiting a longer upper lip than females [17]. In Caucasian populations, the average upper lip length has been reported as approximately 21 mm in males and 20 mm in females. Lip dimensions, including both length and thickness, increase during childhood and adolescence; however, following adolescence, the lips tend to become longer and gradually thinner as part of the ageing process [18,19].

Smile arc

The smile arc refers to the relationship between the curvature formed by the incisal edges of the maxillary anterior teeth and the inner contour of the lower lip during a posed smile. This feature is considered an important determinant of smile aesthetics. A consonant smile arc, regarded as the ideal configuration, is characterized by the curvature of the maxillary incisal edges closely paralleling or coinciding with the contour of the lower lip during smiling. In contrast, a non-consonant smile arc is observed when the incisal edges appear flat or exhibit a reverse curvature relative to the lower lip. Studies have shown that the curvature of the incisal edges is generally more pronounced in women than in men and tends to flatten with advancing age. Similarly, the lower lip typically exhibits greater curvature in younger individuals. The relationship between the lower lip and the maxillary incisors also influences smile attractiveness. The lower lip may lightly touch, remain separate from, or slightly overlap the incisal edges. Evidence suggests that smiles in which the lower lip either touches or does not contact the maxillary incisors are perceived as more aesthetic than those in which the incisors are partially covered by the lower lip [20,21].

## Review

Soft tissue cephalometric analyses for lips used in orthodontics

Steiner’s S Line

Steiner introduced the S line to evaluate lip prominence and profile harmony [22]. This line is obtained by joining the midpoint of the columella (base of nose) to the soft tissue pogonion (chin). The ideal lip position is characterized by the upper and lower lips lying on or slightly behind the S line. Lips ahead of this line indicate a protrusive profile, while much behind suggest a retrusive profile. This assessment is very simple to use and is commonly used in decision-making related to the extraction of teeth in patients having lip protrusion. However, there is a limitation associated that it is dependent on the morphology of the nose and prominence of the chin, and any variations in these can alter the interpretation of the position of the lips.

Ricketts’ E Line (Esthetic Line)

In 1957, Ricketts introduced the esthetic line (E line), which extends from the tip of the nose (pronasale) to the most prominent point of the soft tissue chin (soft tissue pogonion) [2]. Drawn from the tip of the nose (pronasale) to the most prominent point of the chin (soft tissue pogonion), this line enables us to determine lip position (protrusion or retrusion) and facial harmony. In individuals with a well-balanced facial profile, the upper lip is ideally positioned approximately 4 mm posterior to the E line, while the lower lip lies about 2 mm behind the line. All deviations from these values indicate lip protrusion (lips ahead of the E line) or lip retrusion (lips further back). Despite these advantages, one must clearly understand that this analysis is influenced largely by the nasal growth and chin prominence. These factors, in general, may reduce its accuracy, especially in younger patients or in individuals with ethnic variations.

Holdaway’s H Line

Holdaway developed this soft tissue analysis emphasizing lip prominence relative to the chin [3]. The H line is drawn from the soft tissue pogonion to the most prominent point of the upper lip. The H angle, formed between the N-B line and the H line, normally ranges from 7° to 15° (mean approximately 10°). In an aesthetically balanced profile, the lower lip is ideally positioned 0-0.5 mm posterior to the H line, indicating harmonious lip prominence relative to the chin. Holdaway’s analysis has a striking upper hand over other analyses as it focuses on the relationship between the lips and chin rather than relying heavily on nasal morphology. Therefore, it is quite useful in the diagnosis and management of patients with prominent or deficient noses.

Burstone’s B Line

Burstone (1967) introduced the B line to assess lip position independent of nasal prominence [5]. The B line extends from the subnasale to the soft tissue pogonion and serves as a reference for assessing lip protrusion. In an ideal soft tissue profile, the upper lip is positioned approximately 3.5 mm anterior to the B-line, while the lower lip lies about 2.2 mm anterior to it. This enables us to focus more on lip position independent of the nose. This analysis is widely used in orthognathic surgery planning and assessment of lip incompetence. However, the fact that variability in soft tissue thickness and ethnic differences may influence interpretation cannot be overlooked.

Sushner’s S2 Line

Sushner proposed the S2 line to evaluate lip posture in populations with fuller facial profiles [7]. This reference line extends from the soft tissue nasion to the soft tissue pogonion. Lips generally lie anterior to this line. This line is of significance in different ethnic groups and accounts for fuller lip profiles. Clinically, this line assists orthodontists in avoiding over-retraction of incisors and lips during treatment planning in patients with ethnic lip fullness. However, one must consider the fact that individual differences in facial morphology, soft tissue thickness, and changing FA may not be sufficiently taken into consideration by Sushner's S2 line, as it exclusively assesses sagittal lip position.

Nasolabial Angle

This is the angle between the columella of the nose and the upper lip (normal value: 90°-110°), indicating upper lip inclination and is important in extraction decisions. Increased values may indicate upper lip retrusion, whereas decreased values suggest lip protrusion. The nasolabial angle is quite helpful when one is assessing the upper lip inclination and the therapy effects on facial profile. Retraction of incisors as well as extraction of teeth during orthodontic treatment can greatly affect this angle. Age-related changes and nasal morphology may impact this angle, and therefore any interpretation must always be done in conjunction with other analyses as well.

Merrifield’s Z Angle

Merrifield introduced the Z angle to evaluate lower facial balance and chin prominence [8]. The Z angle is formed between the Frankfort horizontal plane and the chin-lower lip line, with a normal value ranging from 70° to 80°. It helps to evaluate lower lip prominence and chin balance. Lower values indicate lip protrusion, while higher values may indicate lip retrusion. This analysis is commonly used in treatment planning that involves orthognathic correction. One of the prime advantages of the Z angle is its ability to integrate chin prominence with lip posture, thereby helping to provide a more comprehensive profile assessment.

Arnett’s True Vertical Line

Arnett introduced a soft tissue cephalometric analysis based on the true vertical line (TVL), a vertical reference line drawn through the subnasale (Sn) with the patient in the natural head position (NHP) [9,23]. In an aesthetically balanced profile, the upper lip anterior (ULA) is positioned approximately 3-4 mm anterior to the TVL, while the lower lip anterior (LLA) lies about 1-2 mm anterior to the line. Lips positioned far ahead of these values indicate lip protrusion, while those positioned behind signify a retrusive or flattened profile. TVL analysis is also used in orthognathic surgery planning as it emphasizes natural head position and soft tissue balance. This analysis is considered highly reliable because it reflects actual facial appearance more accurately than traditional cephalometric planes. However, it is essential to have accurate head positioning when using this analysis to avoid errors.

Comparative evaluation of major soft tissue cephalometric analyses

The major STCA used for the assessment of lip posture and facial profile harmony in ODTP have been summarized in Table 1.

**Table 1:** Major STCA used for the assessment of lip posture and facial profile harmony in orthodontics. STCA: soft tissue cephalometric analyses; TVL: true vertical line.

Analysis	Reference	Lip position/Normal values
Steiner's S Line [22]	Midpoint of columella to soft tissue pogonion	Lips should lie on or slightly behind the line
Ricketts' E Line [2]	Tip of nose to soft tissue pogonion	Upper lip: ~4 mm behind line; lower lip: ~2 mm behind line
Holdaway's H Line [3]	Soft tissue pogonion to upper lip	Lower lip: 0-0.5 mm behind line; H angle: 7-15°
Burstone's B Line [5]	Subnasale to soft tissue pogonion	Upper lip: ~3.5 mm ahead; lower lip: ~2.2 mm ahead
Sushner's S2 Line [7]	Soft tissue nasion to pogonion	Lips generally positioned anterior to line
Nasolabial angle	Columella of nose and upper lip	90-110°
Merrifield's Z Angle [8]	Frankfort horizontal plane and chin-lip line	70-80°
Arnett's TVL [9]	True vertical line through subnasale	Upper lip: 3-4 mm anterior; lower lip: 1-2 mm anterior

Steiner’s S line and Ricketts’ E line are simple and widely used; however, both these analyses are influenced by nasal projection and chin prominence. On the other hand, Holdaway’s H line and Burstone’s B line reduce nasal influence and are thus considered more reliable. While Sushner’s S2 line is valuable in ethnic populations with naturally fuller lips, Arnett’s TVL analysis and Merrifield’s Z angle have proven to be quite beneficial when planning orthognathic surgery cases.

Role of artificial intelligence

Artificial intelligence (AI) has rapidly transformed orthodontic diagnosis and treatment planning by improving the accuracy, efficiency, and predictability of clinical workflows. AI-assisted landmark identification uses deep learning algorithms to automatically detect cephalometric landmarks with high precision, minimizing operator-dependent variability and reducing the time required for analysis. Building on this capability, automated cephalometric tracing enables rapid generation of skeletal, dental, and soft tissue measurements, facilitating accurate diagnosis and treatment planning while enhancing consistency across clinicians. In three-dimensional imaging, AI integrated with cone-beam computed tomography (CBCT) allows soft tissue simulation, enabling clinicians to predict facial profile changes following orthodontic or orthognathic treatment and improve patient communication regarding expected outcomes. Furthermore, AI-enhanced facial scanning provides highly detailed three-dimensional representations of facial morphology, supporting comprehensive evaluation of facial symmetry, soft tissue proportions, and treatment outcomes without exposure to ionizing radiation. More recently, four-dimensional (4D) facial motion analysis has emerged as an advanced application that captures dynamic facial expressions and movements over time, allowing assessment of functional soft tissue behavior during smiling, speaking, and other facial activities. Collectively, these AI-driven technologies are advancing precision orthodontics by integrating static and dynamic facial analyses, improving diagnostic accuracy, personalized treatment planning, and objective evaluation of treatment outcomes [24,25].

Clinical relevance of soft tissue lip analysis

STCA has an important role to play in modern orthodontic treatment planning. Lip posture has a big impact on patient satisfaction, profile harmony, smile aesthetics, and facial attractiveness [26]. Therefore, it is essential that contemporary orthodontic treatment should aim at not only achieving an ideal occlusion but also improving overall facial balance and soft tissue harmony [27].

Also, there has been an increase in aesthetic awareness among patients over the years, and this has made soft tissue evaluation an indispensable component of modern orthodontics [28]. STA are particularly useful when one is juggling between extraction versus non-extraction treatment approaches for managing a case [29]. Patients who are already having lip protrusion, lip incompetence, or excessive facial convexity may benefit from extraction therapy and receive the desired facial profile [30]. On the contrary, in patients with already retrusive lips, if one performs excessive retraction of incisors, this may lead to flattened profiles and finally compromised smile aesthetics. Therefore, a careful assessment of lip posture is essential before deciding treatment mechanics [31].

While planning for orthognathic surgery, STCA definitely has a pivotal role to play in assisting a clinician in predicting post-surgical facial changes. Skeletal movements of the maxilla and mandible directly influence lip support, nasolabial angle, chin prominence, and lower facial harmony. Accurate prediction of these changes can certainly help clinicians achieve superior aesthetic and functional outcomes, thereby enhancing patient satisfaction [32].

Furthermore, all the STAs aid in assessing growth-related facial changes and long-term stability of orthodontic treatment. Growth of the nose, chin, and surrounding soft tissues may alter lip posture over time, thereby affecting FA. Hence, when working on adolescent patients and growing individuals, soft tissue evaluation becomes even more important [33].

Soft tissue lip analyses are also important in camouflage orthodontic treatment, where dental compensations are performed to mask underlying skeletal discrepancies. Evaluation of lip prominence and facial profile helps determine the limits of orthodontic camouflage and prevents undesirable facial changes following excessive incisor movement [34].

The introduction of digital smile designing in modern orthodontics has further enhanced the role of soft tissue analysis while making treatment plans. The digital treatment planning software incorporates soft tissue simulation, enabling clinicians to visualize profile changes and communicate the expected outcomes with patients, thereby increasing the confidence of the patient regarding the success of treatment [35].

The increasing emphasis on personalized orthodontics has further highlighted the importance of soft tissue evaluation [36].

Future perspectives

The future lies in AI-assisted STCA and machine learning algorithms, which are likely to be integrated into orthodontic diagnosis and digital treatment planning in times to come [37]. The use of 3D imaging techniques has definite potential in enhancing the evaluation of soft tissue thickness, facial symmetry, and dynamic smile analysis [38]. Future ODTP is expected to become more individualized, combining skeletal, dental, and soft tissue parameters in accordance with patient-specific aesthetic goals.

## Conclusions

Soft tissue lip analysis is an essential component of ODTP. Various analyses such as Steiner’s S line, Ricketts’ E line, Holdaway’s H line, Burstone’s B line, Sushner’s S2 line, Merrifield’s Z angle, nasolabial angle, and Arnett’s TVL provide valuable information regarding FA and lip posture. However, no single analysis should be used as a standalone method. Orthodontists must utilize more than one soft tissue analysis and correlate the findings with clinical evaluation to achieve optimal aesthetic and functional outcomes.
